# The Rational Use of Disinfectants

**Published:** 1907-02-02

**Authors:** 


### Editor's Letter-Box.

### "THE RATIONAL USE OF DISINFECTANTS."

*To the Editor of* The Hospital.

Sir,—In reference to the above very interesting article, which appeared in your issue of January 5, we feel sure you will allow us to make a few comments.

In referring to Izal disinfectant, Dr. Divine remarks that we have followed Jeyes' Company recently in guaranteeing a germicidal power in definite and scientific terms. We think Dr. Divine is under a misapprehension. If he suggests that we have adopted the pure culture method of standardisation as formulated by Dr. S. Rideal and Mr. A. Walker, managing director of Jeyes' Company, he is in error, for we hold with Mr. Winter-Blyth, Professor Kenwood, and most advanced experts, that this "pure-culture" method is quite useless in standardising disinfectants which will be used in impure media. If, on the other hand, he suggests that we were later than Jeyes in advocating bacteriological in preference to chemical examination for disinfectants, the exact reverse is the case. Bacteriological work was done with Izal at our suggestion, even before 1892, in which year was published Professor Klein's report on its behaviour towards a variety of disease germs. At this time, and for many years later, we know of no other firm of disinfectant manufacturers who expressed the slightest interest in bacteriological examination. Messrs. Jeyes were, indeed, circulating a pamphlet entitled "Facts About Disinfectants," by Hebling and Passmore, which strongly upheld the chemical as against the bacteriological method. Bacteriological examination has for over fifteen years been a foremost principle with us, and we hope before long that scientific experts of standing will provide us with an adequate method of standardisation. Such a method must, in our opinion, compare disinfectants under circumstances as nearly as possible akin to those met with in practice.

Yours faithfully,
Newton, Chambers and Co., Limited.
The Laboratories, Thorncliffe, near Sheffield.
January 15, 1907.

